# Supplementary material for: Membrane thickness, lipid phase and sterol type are determining factors in the permeability of membranes to small solutes
Source: Nat Commun. 2022 Mar 25;13:1605. doi: 10.1038/s41467-022-29272-x (PMC8956743; doi:10.1038/s41467-022-29272-x)
Supplement: Supplementary file 1 — Supplementary Information [file 41467_2022_29272_MOESM1_ESM.pdf]

SUPPLEMENTARY INFORMATION

# Membrane thickness, lipid phase and sterol type are determining factors in the permeability of membranes to small solutes

Jacopo Frallicciardi[1], Josef Melcr[2], Pareskevi Siginou[1], Siewert J. Marrink[2] and Bert Poolman[1]

Departments of Biochemistry[1] and Biophysical Chemistry[2]
University of Groningen
Nijenborgh 4, 9747 AG Groningen, the Netherlands

Correspondence to b.poolman@rug.nl or s.j.marrink@rug.nl
JF and JM share first-authorship on this paper

**Keywords:** Membrane permeability, passive diffusion, physical state of membrane, pre-steady state kinetics, molecular dynamics simulations

# Supplementary Figures

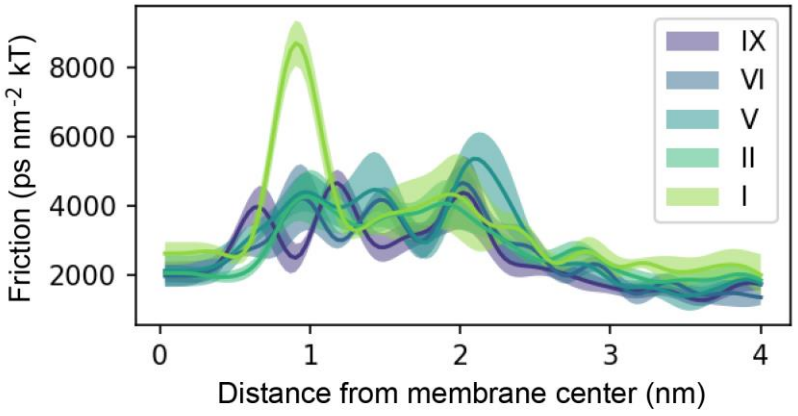


**Supplementary Figure 1. Friction profiles of solutes of different hydrophobicity as a function of the distance from membrane center (DOPC bilayer).** More hydrophilic solutes (*i.e.*, level I) exhibit larger friction at the region, where the permeating solute desolvates (around 1 nm). Error estimates represented by shaded areas around the mean curves are described in Methods. Briefly, the error estimate was calculated from the average noise around the denoised curve and from the deviations from the symmetrical shape between the left and right part of the profile.

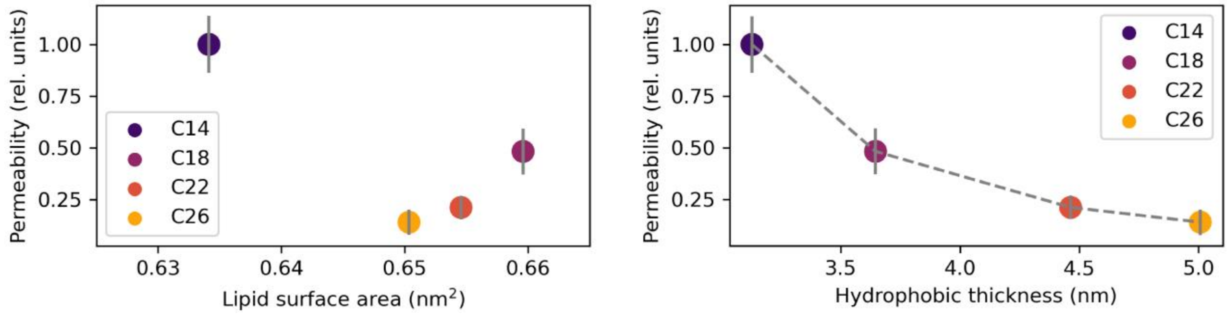


**Supplementary Figure 2. Permeability coefficients as a function of lipid surface area (left) and the membrane hydrophobic thickness (right).** Permeability coefficients are normalized to the membrane with the lowest thickness (C14). Data are presented as mean values +/- SEM. Error estimates represented by error bars around the mean values are described in Methods ("Inhomogeneous solubility-diffusion model"). Lipids with mono-unsaturated tails of lengths between 14 and 26 carbons correspond to 1,2-dimyristoleoyl-*sn*-glycero-3-phosphocholine (C=14), 1,2-dioleoyl-*sn*-glycero-3-phosphocholine (C=18), 1,2-dierucoyl-*sn*-glycero-3-phosphocholine (C=22) and 1,2-dihexacosenoyl-*sn*-glycero-3-phosphocholine (C=26), respectively. While the area per lipid is found to correlate with the permeability coefficient in previous work[1], our simulations do not reveal such correlations. Instead, the expected correlation with thickness is recovered in both experiments and simulations (also in Fig. 2 in the main text).

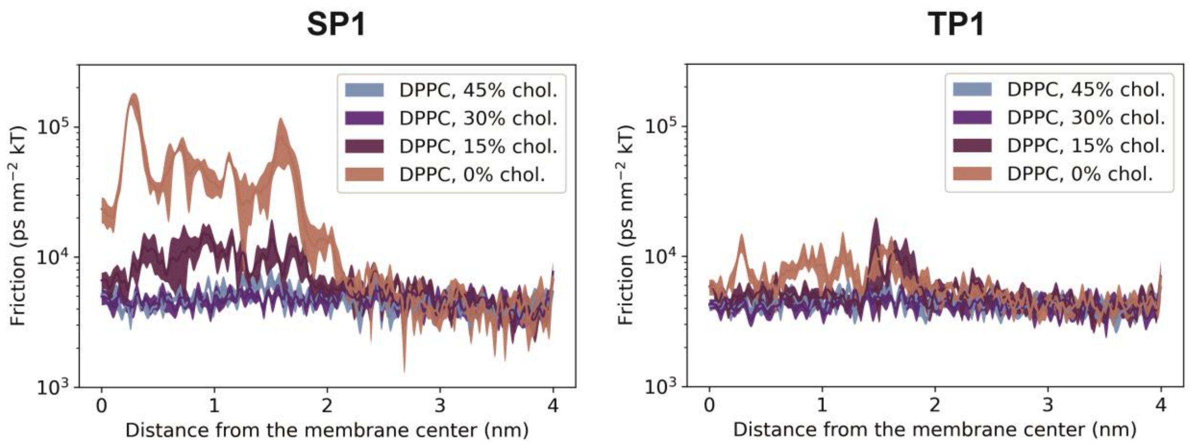


**Supplementary Figure 3. Friction profiles from MD simulations with particles at the hydrophobicity level III of different sizes - “small” SP1 (size ~ 3 water molecules) and “tiny” TP1 (size ~ 2 water molecules)**. The larger solute, SP1, has lower diffusivity through the membranes at a $L_\beta$ phase (DPPC bilayer). Adding 15 mol% of cholesterol to the DPPC membrane leads to a decrease of the friction due to the perturbed packing of the phospholipid tails by the sterols. Such an effect is not as significant for smaller solutes as seen from the changes in the friction profiles from the TP1 solute.

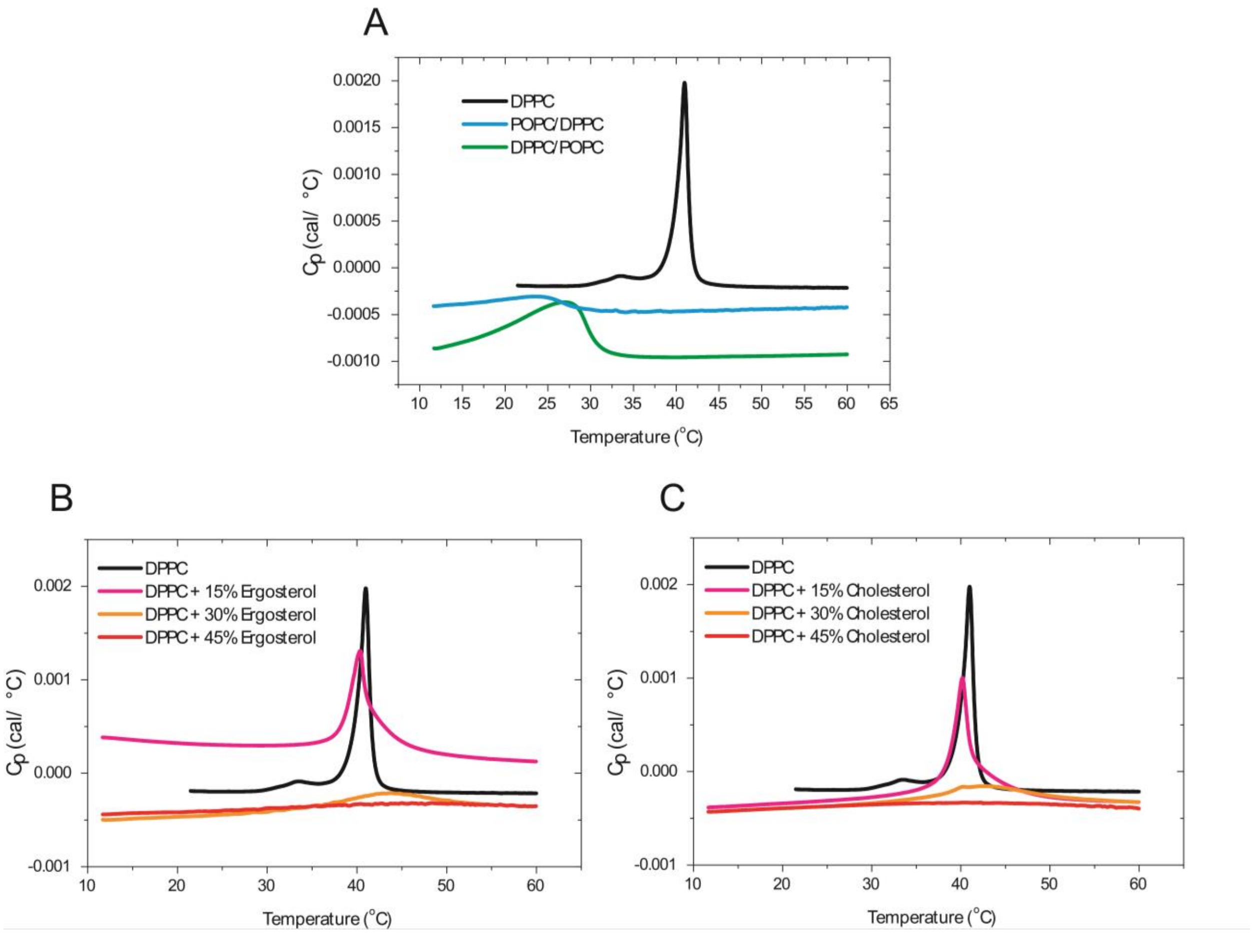


**Supplementary Figure 4. Normalized DSC endotherms of lipid vesicle solutions.** The assay buffer (100 mM KPi buffer pH 7.0) is used as baseline. A. Endotherms of pure DPPC, POPC/DPPC (ratio 67:33), and DPPC/POPC (ratio 67:33) vesicles. B. Endotherms of pure DPPC vesicles and DPPC + cholesterol vesicles in the ratio of 85:15, 70:30 and 55:45. C. Endotherms of pure DPPC vesicles and DPPC + ergosterol vesicles in the ratio of 85:15, 70:30 and 55:45.

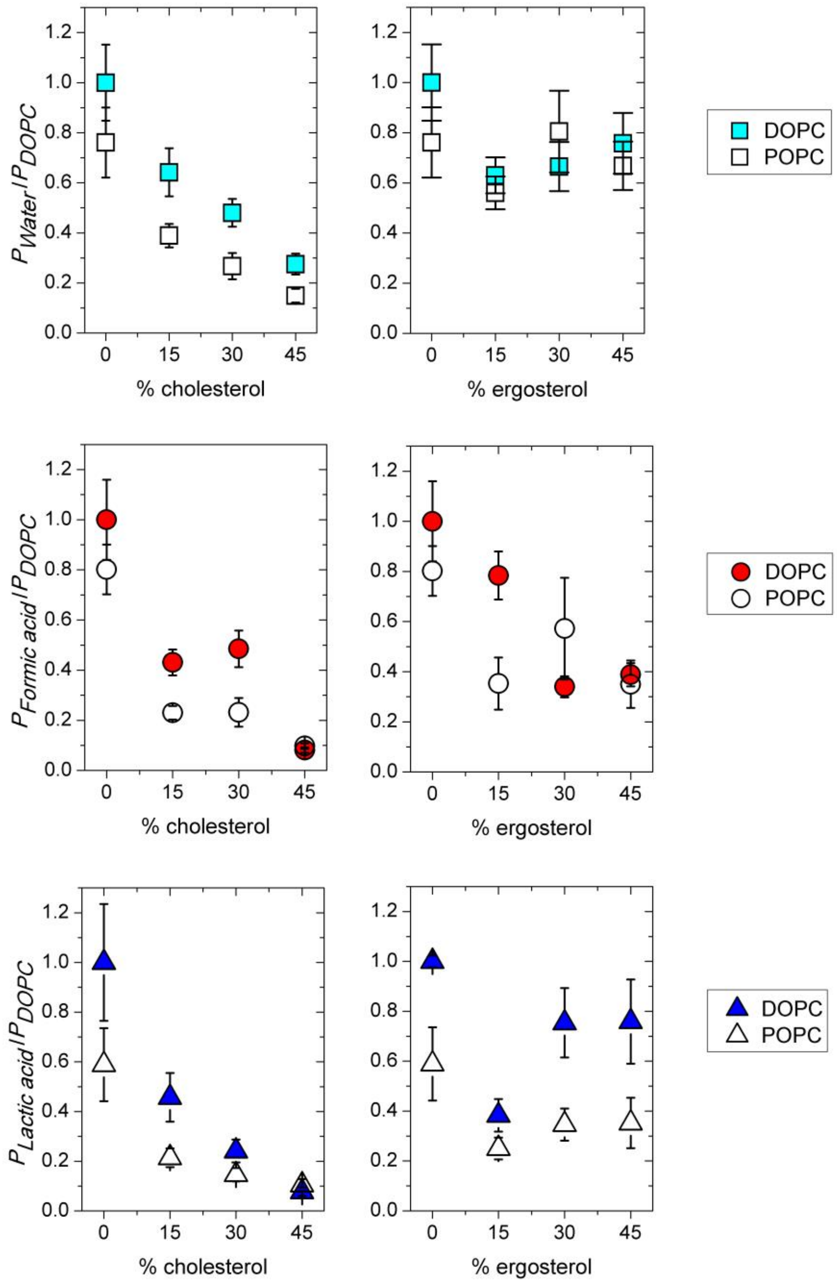


**Supplementary Figure 5. Relative permeability of DOPC and POPC vesicles as a function of cholesterol (left) and ergosterol (right) content.** Permeability from experiments at 20 °C of water (squares), formic acid (circles) and lactic acid (triangles) as a function of sterol content in vesicles composed of pure DOPC and POPC, which are represented by filled and hollow markers, respectively. Permeability coefficients $P$ (cm/s) are normalized to the value of pure DOPC. The permeability coefficient in DOPC vesicles was 16.0 (±1.7) x $10^{-3}$ cm/s, 6.73 (±0.74) x $10^{-3}$ cm/s, and 0.198 (±0.033) x $10^{-3}$ cm/s for water, formic acid, and L-lactic acid, respectively. Data are presented as mean values +/- SEM. Error estimates represented by error bars around the mean values are described in Methods ("Fit of the in vitro kinetics").

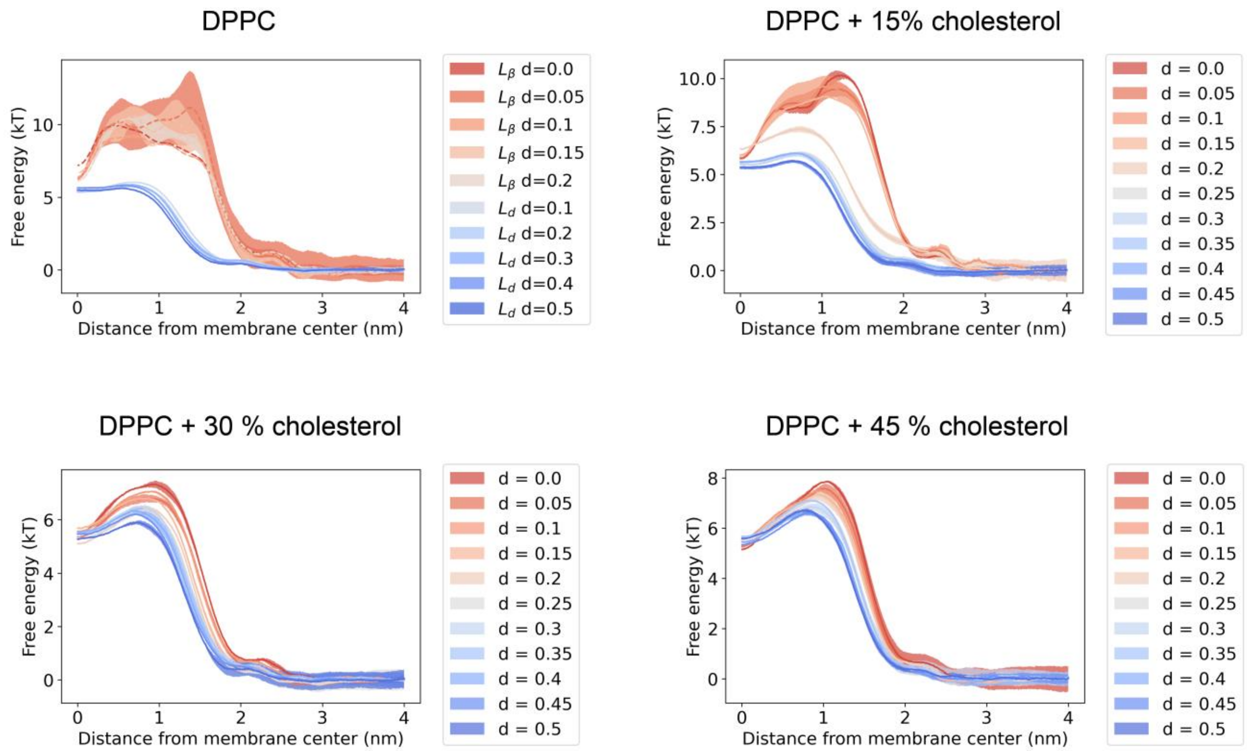


**Supplementary Figure 6. Free energy profiles from simulated membranes with 0, 15, 30 and 45 mol% cholesterol and a variable unsaturation index *d*.** The profiles smoothly change from the composition with POPC (blue, *d*=0.5) to that with DPPC (red, *d*=0.0) within a single phase, but exhibit large non-smooth changes with phase transitions affecting the permeability coefficients.

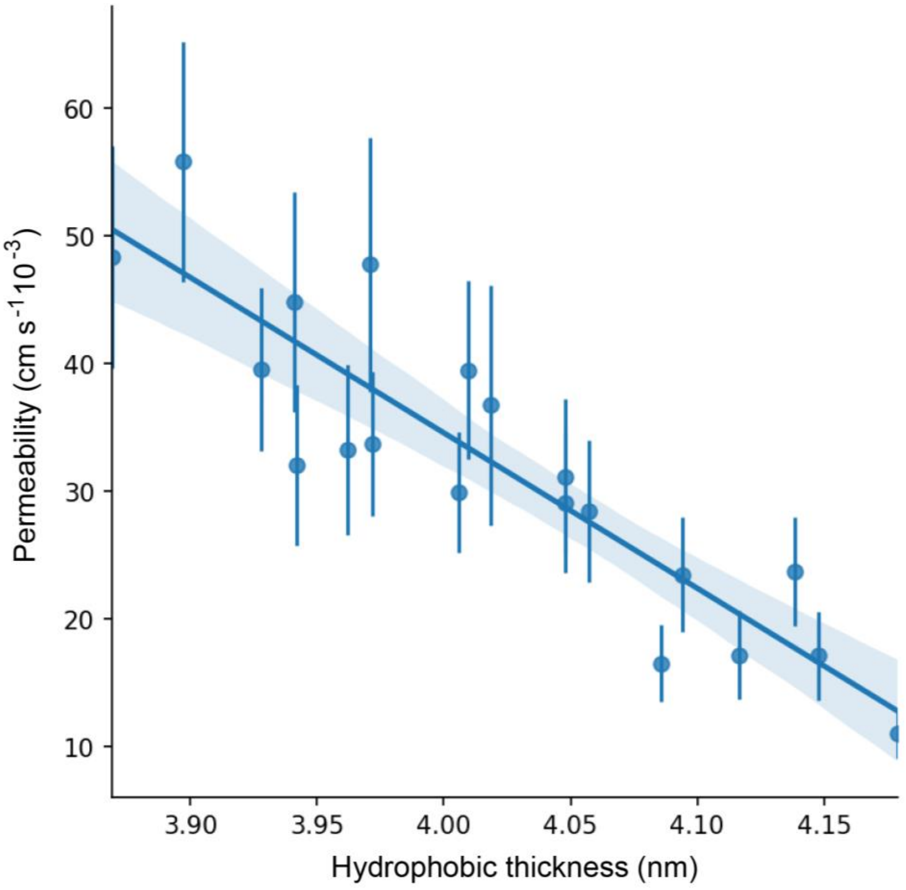


**Supplementary Figure 7. Regression fit of the permeability as a function of the membrane hydrophobic thickness.** Data points are from simulated membranes with a varying degree of tail saturation (index between 0.35 and 0.5) and sterol concentrations (between 0 and 45 mol%). Shaded area denotes the 95% confidence interval. Data are presented as mean values +/- SEM. Error estimates represented by error bars around the mean values are described in Methods ("Inhomogeneous solubility-diffusion model").

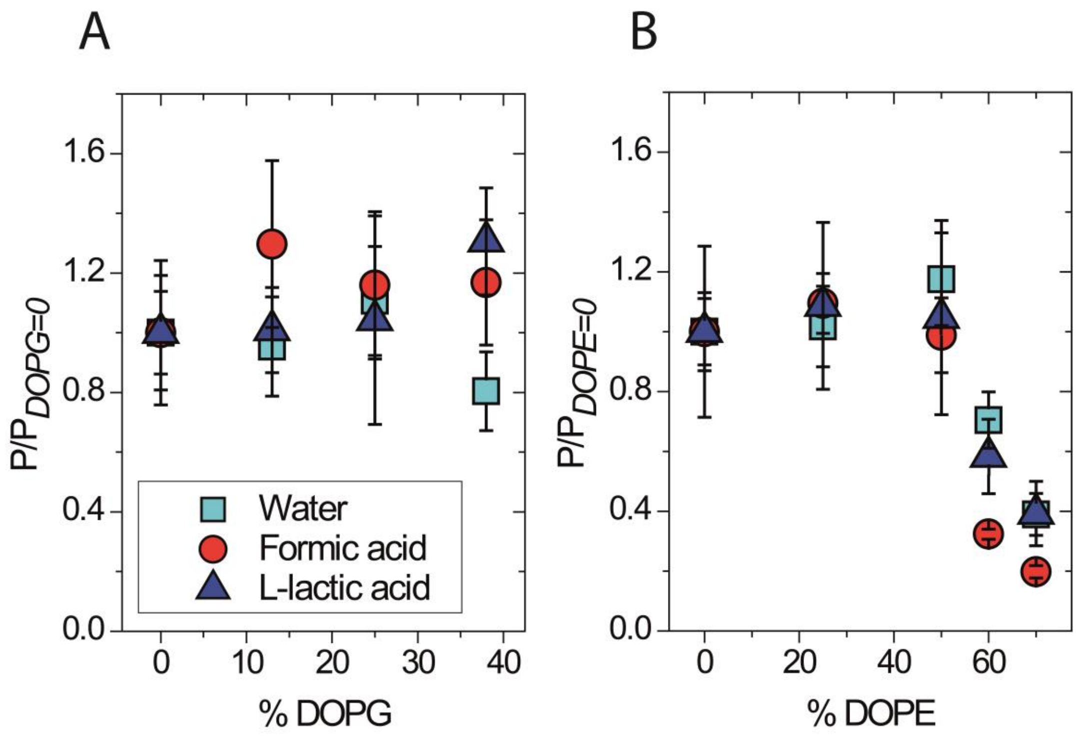


**Supplementary Figure 8. Permeability from experiments at 20 °C of water (cyan squares), formic acid (red circles), and L-lactic acid (blue triangles) as a function of lipid head group composition.** A. DOPG titration. The fraction of DOPE was kept constant at 50% and DOPG was varied reciprocally with DOPC. Permeability coefficients are normalized to $P_{DOPG=O}$. The permeability coefficient in $P_{DOPG=O}$ vesicles was 13.8 (±1.9) x $10^{-3}$ cm/s, 6.37 ± (1.09) x $10^{-3}$ cm/s, and 0.116 (±0.016) x $10^{-3}$ cm/s for water, formic acid, and lactic acid, respectively. B. DOPE titration. The fraction of DOPG was kept constant at 25%, and DOPE was varied reciprocally with DOPC. Permeability coefficients are normalized to $P_{DOPE=O}$. The permeability coefficient in $P_{DOPE=O}$ vesicles was 13.0 (±1.2) x $10^{-3}$ cm/s, 7.47 ± (0.83) x $10^{-3}$ cm/s, and 0.119 (±0.024) x $10^{-3}$ cm/s for water, formic acid, and lactic acid, respectively. Data are presented as mean values +/- SEM. Error estimates represented by error bars around the mean values are described in Methods ("Fit of the in vitro kinetics"). The numerical values of the permeability coefficients are presented in Supplementary Table 5.

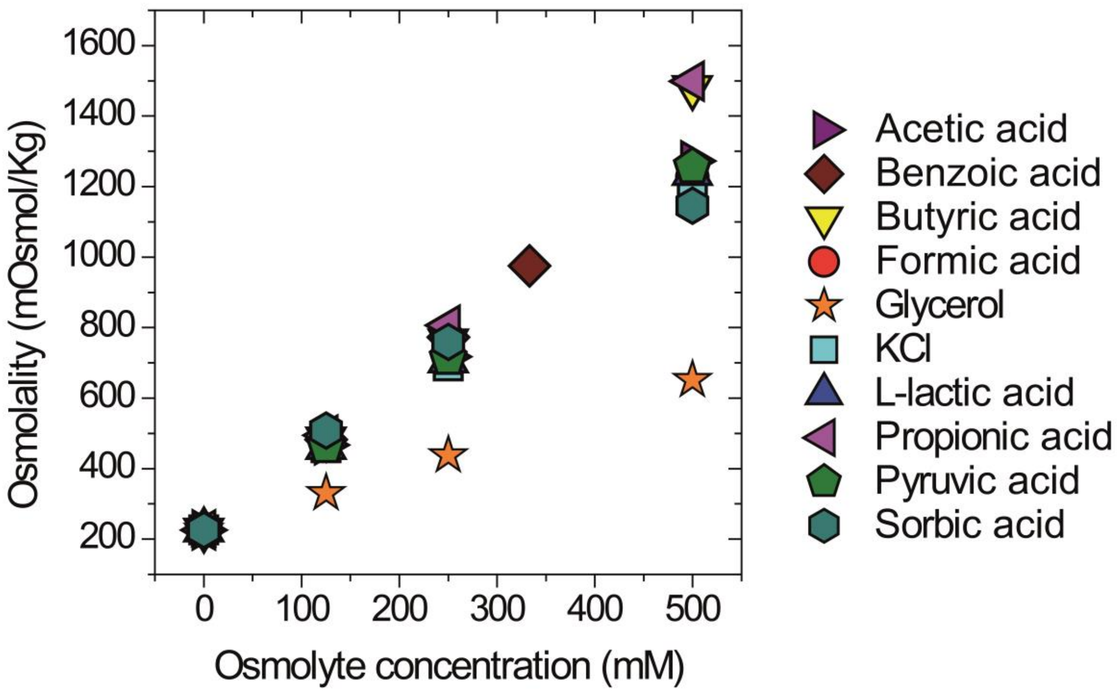


**Supplementary Figure 9. Plots of the measured osmolality as a function of the osmolyte concentration.** The data were fitted with a linear relation (y = mx + q) that was later used to prepare the osmolyte solutions at the desired osmolality of ca. 300 mosmol/kg. The following slopes (m) were used for the calculations: 0.852 (glycerol), 1.822 (K-sorbate), 1.910 (KCl), 2.102 (Na-acetate), 2.367 (Na-benzoate), 2.528 (Na-butyrate), 2.001 (Na-formate), 2.030 (Na-L-lactate), 2.568 (Na-propionate) and 2.068 (Na-pyruvate). The intercept was q = 225.5, which corresponds to the osmolality of the assay buffer (100 mM potassium phosphate, pH 7.0).

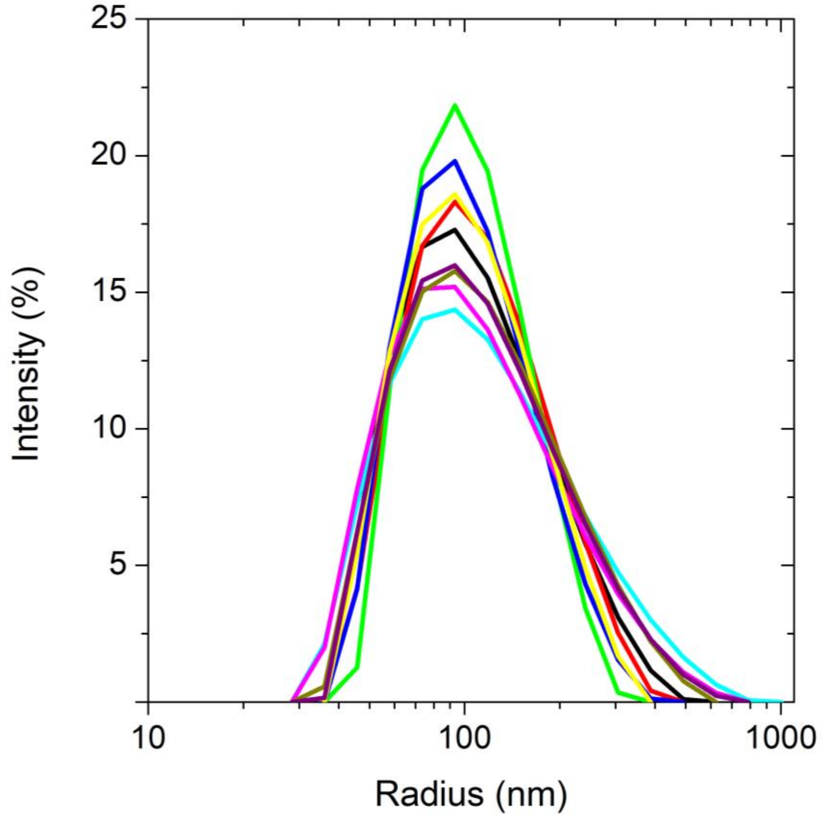


**Supplementary Figure 10**. **Size distributions measured with DLS of DOPC vesicles extruded through a 200 nm polycarbonate filter.** Ten overlaid acquisitions are shown.

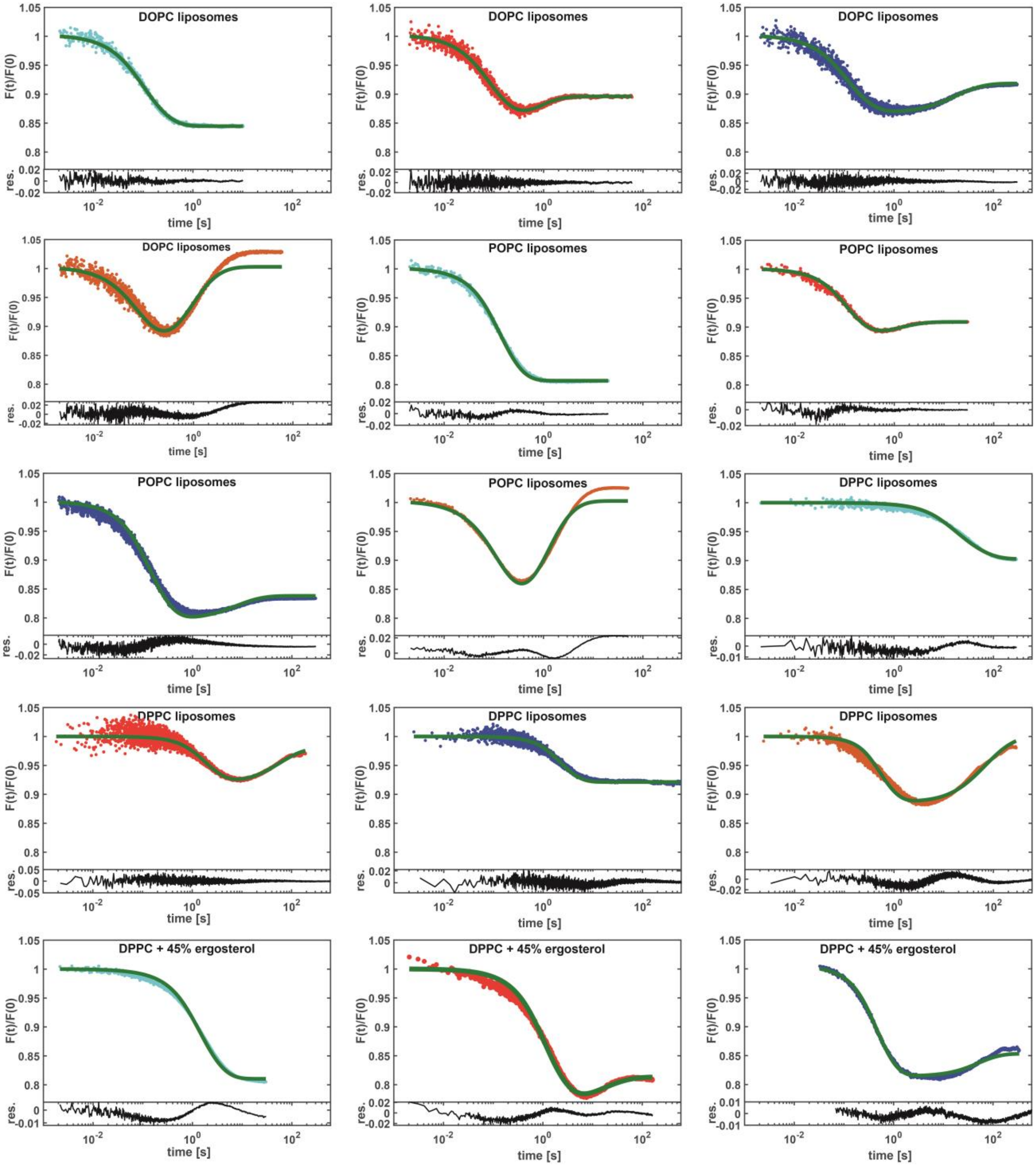


**Supplementary Figure 11**. **Fits of the calcein relaxation curves for vesicles composed of pure DOPC, pure POPC, pure DPPC, and DPPC + 45% ergosterol.** Osmotic upshift with KCl in cyan, with Na-formate in red, with Na-Lactate in dark blue, and glycerol in orange. The residuals (res.) are given in the bottom panel. The fitting curve is shown in green. The fitting parameters are given in Supplementary Table 6.

# Supplementary Tables

| Compound | Log$P_{OW}$ | Experimental permeability coefficients x10$^{-3}$ (cm s$^{-1}$) |
|---|---|---|
| Water | - | 16.0 ± 1.7 |
| Glycerol | -1.76 | 0.023 ± 0.002 |
| L-lactic acid | -0.72 | 0.198 ± 0.033 |
| Formic acid | -0.54 | 6.73 ± 0.74 |
| Pyruvic acid | -0.5 | 1.20 ± 0.17 |
| Acetic acid | -0.17 | 95.1 ± 1.1 |
| Propionic acid | 0.33 | 101 ± 3 |
| Butyric acid | 0.79 | 214 ± 34 |
| Sorbic acid | 1.33 | 291 ± 30 |
| Benzoic acid | 1.87 | 336 ± 51 |

| Hydrophobicity level and simulated particle name | Log$P_{OW}$ | Log$P_{MW}$ | Calculated permeability coefficients x10$^{-3}$ (cm s$^{-1}$) |
|---|---|---|---|
| I, SP6 | -2.14 | -2.03 | 0.17 ± 0.07 |
| II, SP3 | -1.35 | -1.15 | 35 ± 2 |
| III, SP1 | -0.91 | -0.88 | 96 ± 11 |
| IV, SN5 | -0.63 | -0.66 | 402 ± 80 |
| V, SN3 | -0.32 | -0.33 | 2050 ± 200 |
| VI, SN2 | 0.37 | -0.06 | 7700 ± 800 |
| VII, SN1 | 0.63 | 0.11 | 10400 ± 1500 |
| VIII, SC6 | 0.93 | 0.26 | 12800 ± 2000 |
| IX, SC5 | 1.1 | 0.46 | 11600 ± 2000 |

**Supplementary Table 1**. Permeability coefficients in lipid vesicles composed of pure DOPC and octanol/water, and membrane/water partitioning coefficient (log$P_{OW}$, log$P_{MW}$) from experiments at 20 °C and simulations. Log$P_{OW}$ values for weak acids and glycerol were taken from the PubChem database (https://pubchem.ncbi.nlm.nih.gov/).

| Lipids | Acyl chain length | $P_{Water}$ x10$^{-3}$ (cm/s) | $P_{formic\ acid}$ x10$^{-3}$ (cm/s) | $P_{lactic\ acid}$ x10$^{-3}$ (cm/s) | $P_{MD}$ x10$^{-3}$ (cm/s) |
|---|---|---|---|---|---|
| (14:1) PC | 14 | 22.8 ± 1.9 | 11.80 ± 1.43 | 0.332 ± 0.052 | 199 ± 30 |
| (16:1) PC | 16 | 16.3 ± 1.1 | 8.31 ± 0.64 | 0.196 ± 0.012 | / |
| (18:1) PC/DOPC | 18 | 16.0 ± 1.7 | 6.73 ± 0.74 | 0.198 ± 0.033 | 96 ± 11 |
| (20:1) PC | 20 | 7.1 ± 0.9 | 3.39 ± 0.13 | 0.098 ± 0.011 | / |
| (22:1) PC | 22 | 5.9 ± 0.6 | 1.90 ± 0.22 | 0.078 ± 0.010 | 42 ± 2 |
| (26:1) PC | 26 | / | / | / | 28 ± 2 |

**Supplementary Table 2.** Permeability coefficients at 20 °C for formic acid, lactic acid, water, and the simulated solute of hydrophobicity level III (SP1) in lipid vesicles composed of pure PC with varying acyl chain length from experiments and simulations.

| Mixture | d | Expected phase | $P_{water}$ x10$^{-3}$ (cm/s) | $P_{formic\ acid}$ x10$^{-3}$ (cm/s) | $P_{lactic\ acid}$ x10$^{-3}$ (cm/s) | $P_{glycerol}$ x10$^{-5}$ (cm/s) | $P_{MD}$ x10$^{-3}$ (cm/s) |
|---|---|---|---|---|---|---|---|
| DOPC | 1 | $L_d$ | 16.0 ± 1.7 | 6.73 ± 0.74 | 0.198 ± 0.033 | 2.3 ± 0.2 | 96 ± 11 |
| DOPC/POPC | 0.84 | $L_d$ | 13.5 ± 0.5 | 6.04 ± 0.36 | 0.177 ± 0.021 | / | / |
| POPC/DOPC | 0.67 | $L_d$ | 12.3 ± 1.5 | 5.68 ± 0.48 | 0.153 ± 0.031 | / | / |
| POPC | 0.5 | $L_d$ | 12.0 ± 1.4 | 5.53 ± 0.74 | 0.117 ± 0.022 | 1.27 ± 0.1 | 48 ± 9 |
| POPC/DPPC | 0.34 | *$L_d$ + $L_\beta$ | 8.7 ± 1.0 | 4.90 ± 0.92 | 0.083 ± 0.008 | / | 33 ± 7 |
| DPPC/POPC | 0.17 | *$L_d$ + $L_\beta$ | 5.9 ± 0.3 | 2.36 ± 0.56 | 0.049 ± 0.004 | / | 31 – 0.08 |
| DPPC | 0 | * $L_\beta$ | 0.078 ± 0.010 | 0.0027 ± 0.0005 | Not observed | 0.00011 ± 0.00002 | 0.06 ± 0.02 |

**Supplementary Table 3.** Permeability coefficients at 20 °C for various compounds in lipid vesicles composed of pure DOPC, POPC and DPPC, and mixtures of DOPC/POPC and POPC/DPPC from experiments and simulations. Parameter *d* represents the degree of unsaturation. The errors (SD) originate from the inaccuracy of the vesicle size distribution. *****Mixtures analyzed by DSC (Supplementary Fig. S4A).

| Lipid mixture | Expected phase | $P_{water}$ x10$^{-3}$ (cm/s) | $P_{formic\ acid}$ x10$^{-3}$ cm/s) | $P_{lactic\ acid}$ x10$^{-3}$ (cm/s) | $P_{MD}$ x10$^{-3}$ (cm/s) |
|---|---|---|---|---|---|
| DOPC | $L_d$ | 16.0 ± 1.7 | 6.73 ± 0.74 | 0.198 ± 0.033 | 96 ± 11 |
| DOPC + 15 % Chol | $L_d$ | 10.3 ± 1.1 | 3.10 ± 0.12 | 0.090 ± 0.012 | / |
| DOPC + 30 % Chol | $L_O$ | 7.69 ± 0.41 | 3.49 ± 0.35 | 0.047 ± 0.004 | / |
| DOPC + 45 % Chol | $L_O$ | 4.41 ± 0.47 | 0.58 ± 0.04 | 0.015 ± 0.002 | / |
| DOPC + 15 % Erg | $L_d$ | 10.1 ± 0.4 | 5.64 ± 0.26 | 0.076 ± 0.002 | / |
| DOPC + 30 % Erg | $L_O$ | 10.6 ± 1.1 | 2.45 ± 0.13 | 0.149 ± 0.012 | / |
| DOPC + 45 % Erg | $L_O$ | 12.1 ± 1.4 | 2.79 ± 0.11 | 0.150 ± 0.022 | / |
| POPC | $L_d$ | 12.0 ± 1.4 | 5.53 ± 0.74 | 0.117 ± 0.022 | 48 ± 9 |
| POPC + 15 % Chol | $L_d$ | 6.22 ± 0.34 | 1.65 ± 0.49 | 0.042 ± 0.002 | 32 ± 6 |
| POPC + 30 % Chol | $L_d$ + $L_O$ | 4.28 ± 0.70 | 1.67± 0.36 | 0.029 ± 0.002 | 37 ± 9 |
| POPC + 45 % Chol | $L_O$ | 2.38 ± 0.36 | 0.70 ± 0.02 | 0.021 ± 0.003 | 17 ± 3 |
| POPC + 15 % Erg | $L_d$ | 8.97 ± 0.40 | 2.54 ± 0.69 | 0.049 ± 0.003 | / |
| POPC + 30 % Erg | $L_d$ + $L_O$ | 12.9 ± 2.2 | 4.11 ± 1.38 | 0.068 ± 0.005 | / |
| POPC + 45 % Erg | $L_O$ | 10.7 ± 1.0 | 2.53 ± 0.62 | 0. 070 ± 0.002 | / |
| DPPC | *$L_\beta$ | 0.078 ± 0.010 | 0.0027 ± 0.0005 | Not observed | 0.06 ± 0.02 |
| DPPC + 15 % Chol | *$L_\beta$ + $L_O$ | 0.23 ± 0.04 | 0.041 ± 0.003 | Not observed | 0.3 ± 0.1 |
| DPPC + 30 % Chol | *$L_\beta$ + $L_O$ | 0.33 ± 0.02 | 0.046 ± 0.003 | Not observed | 8 ± 1 |
| DPPC + 45 % Chol | *$L_O$ | 0.46 ± 0.07 | 0.031 ± 0.008 | Not observed | 6 ± 2 |
| DPPC + 15 % Erg | * $L_\beta$ + $L_O$ | 0.12 ± 0.02 | 0.013 ± 0.001 | Not observed | / |
| DPPC + 30 % Erg | * $L_\beta$ + $L_O$ | 0.54 ± 0. 05 | 0.056 ± 0.003 | 0.0013 ± 0.0003 | / |
| DPPC + 45% Erg | *$L_O$ | 0.86 ± 0.07 | 0.096 ± 0.006 | 0.0023 ± 0.0003 | / |

**Supplementary Table 4.** Permeability coefficients at 20 °C for various compounds in lipid vesicles composed of pure DOPC, POPC and DPPC with cholesterol or ergosterol from experiments and simulations. ***Mixtures analyzed by DSC (Supplementary Fig. S4B and SC).

| Lipid mixture | Ratio | $P_{water}$ x10$^{-3}$ (cm/s) | $P_{formic\ acid}$ x10$^{-3}$ cm/s) | $P_{lactic\ acid}$ x10$^{-3}$ (cm/s) |
|---|---|---|---|---|
| DOPE:DOPC:DOPG | 0:75:25 | 13.0 ± 1.2 | 7.47 ± 0.83 | 0.119 ± 0.024 |
| DOPE:DOPC:DOPG | 25:50:25 | 13.2 ± 1.3 | 8.18 ± 0.75 | 0.151 ± 0.020 |
| DOPE:DOPC:DOPG | 50:25:25 | 15.3 ± 1.4 | 7.38 ± 0.93 | 0.121 ± 0.040 |
| DOPE:DOPC:DOPG | 60:15:25 | 9.2 ± 0.9 | 2.42 ± 0.13 | 0.069 ± 0.005 |
| DOPE:DOPC:DOPG | 70:05:25 | 5.1 ± 1.5 | 1.48 ± .015 | 0.047 ± 0.009 |
| DOPE:DOPC:DOPG | 50:50:0 | 13.8 ± 1.9 | 6.37 ± 1.09 | 0.116 ± 0.016 |
| DOPE:DOPC:DOPG | 50:37:13 | 13.2 ± 1.4 | 8.26 ± 1.08 | 0.117 ± 0.016 |
| DOPE:DOPC:DOPG | 50:12:38 | 11.1 ± 1.0 | 7.44 ± 0.40 | 0.151 ± 0.021 |

**Supplementary Table 5.** Permeability coefficients at 20 °C for water, formic acid, and lactic acid in liposomes composed of mixtures of DOPE:DOPC:DOPG or POPE:POPC:POPG in different ratios from experiments.

| Lipid mixture | Osmolyte | Parameter 1 [$K_{SV}$] | Parameter 2 [$P_{water}$ (cm/s)] | Parameter 3 [$P_{osmolyte}$ (cm/s)] |
|---|---|---|---|---|
| DOPC | KCl | 59753 | 1.7971 x$10^{-2}$ | 0 (fixed) |
| DOPC | Na-Formate | 68555 | 1.5265 x$10^{-2}$ | 7.0563 x$10^{-3}$ |
| DOPC | Na-Lactate | 21440 | 1.5736 x$10^{-2}$ | 1.6141 x$10^{-4}$ |
| DOPC | Glycerol | 143820 | 5.3825 x$10^{-3}$ | 2.5822 x$10^{-5}$ |
| POPC | KCl | 29934 | 1.1904 x$10^{-2}$ | 0 (fixed) |
| POPC | Na-Formate | 41411 | 1.2157 x$10^{-2}$ | 6.2516 x$10^{-3}$ |
| POPC | Na-Lactate | 34347 | 8.5187 x$10^{-3}$ | 1.375 x$10^{-4}$ |
| POPC | Glycerol | 297210 | 7.6111 x$10^{-3}$ | 1.1509 x$10^{-5}$ |
| DPPC | KCl | 25526 | 7.7376 x$10^{-5}$ | 0 (fixed) |
| DPPC | Na-Formate | 18468 | 1.0869 x$10^{-4}$ | 2.2099 x$10^{-7}$ |
| DPPC | Na-Lactate | 20691 | 6.9447 x$10^{-5}$ | 0 (fixed) |
| DPPC | Glycerol | 12006 | 1.6695 x$10^{-4}$ | 8.8725 x$10^{-10}$ |
| DPPC + 45% Ergosterol | KCl | 30600 | 1.0325 x$10^{-3}$ | 0 (fixed) |
| DPPC + 45% Ergosterol | Na-Formate | 43496 | 1.2393 x$10^{-3}$ | 1.2153 x$10^{-4}$ |
| DPPC + 45% Ergosterol | Na-Lactate | 30663 | 8.7443 x$10^{-4}$ | 2.3123 x$10^{-5}$ |

**Supplementary Table 6.** Parameters generated by the fitting of the calcein relaxation curves for liposomes of pure DOPC, pure POPC, pure DPPC, and DPPC + 45% ergosterol. The reported parameters refer to an exemplary fitting performed by using only one liposome size distributions. The relative fits are shown in Supplementary Figure S11.

## Supplementary References

1. Mathai, J. C., Tristram-Nagle, S., Nagle, J. F. & Zeidel, M. L. Structural Determinants of Water Permeability through the Lipid Membrane. *J. Gen. Physiol.* **131**, 69–76 (2008).
