# Supplementary material for: Membrane thickness, lipid phase and sterol type are determining factors in the permeability of membranes to small solutes
Source: Nat Commun. 2022 Mar 25;13:1605. doi: 10.1038/s41467-022-29272-x (PMC8956743; doi:10.1038/s41467-022-29272-x)
Supplement: Supplementary file 3 — Reporting Summary [file 41467_2022_29272_MOESM3_ESM.pdf]

nature portfolio

Corresponding author(s): Bert Poolman, Siewert-Jan Marrink

Last updated by author(s): Feb 2, 2022

Reporting Summary

Nature Portfolio wishes to improve the reproducibility of the work that we publish. This form provides structure for consistency and transparency in reporting. For further information on Nature Portfolio policies, see our Editorial Policies and the Editorial Policy Checklist.

Statistics

For all statistical analyses, confirm that the following items are present in the figure legend, table legend, main text, or Methods section.

n/a	Confirmed	
☐	☒	The exact sample size (*n*) for each experimental group/condition, given as a discrete number and unit of measurement
☒	☐	A statement on whether measurements were taken from distinct samples or whether the same sample was measured repeatedly
☒	☐	The statistical test(s) used AND whether they are one- or two-sided<br>*Only common tests should be described solely by name; describe more complex techniques in the Methods section.*
☒	☐	A description of all covariates tested
☒	☐	A description of any assumptions or corrections, such as tests of normality and adjustment for multiple comparisons
☐	☒	A full description of the statistical parameters including central tendency (e.g. means) or other basic estimates (e.g. regression coefficient) AND variation (e.g. standard deviation) or associated estimates of uncertainty (e.g. confidence intervals)
☒	☐	For null hypothesis testing, the test statistic (e.g. *F*, *t*, *r*) with confidence intervals, effect sizes, degrees of freedom and *P* value noted<br>*Give P values as exact values whenever suitable.*
☒	☐	For Bayesian analysis, information on the choice of priors and Markov chain Monte Carlo settings
☒	☐	For hierarchical and complex designs, identification of the appropriate level for tests and full reporting of outcomes
☒	☐	Estimates of effect sizes (e.g. Cohen's *d*, Pearson's *r*), indicating how they were calculated

Our web collection on statistics for biologists contains articles on many of the points above.

Software and code

Policy information about availability of computer code

Data collection	Built-in softwares Pro Data SX, Pro-Data viewer, and APL Pro-Data Converter for data collection with a stopped-flow apparatus (SX20; Applied Photophysics, Leatherhead, Surrey, UK). Built-in software DYNAMICS for DynaPro NanoStar Detector (Wyatt Technology, Santa Barbara, CA). ORIGIN software provided by MicroCal (Amherst, MA) for differential scanning calorimetry (DSC). GROMACS molecular simulation suite (open source).
Data analysis	MATLAB software (R2018a). FMINUIT for MATLAB (open source). Python (open source). Seaborn python library (open source). Numpy python standard library (open source). Pandas python library (open source). Scripts used to analyze experimental data and calculate permeability coefficients are available in an open public repository (doi.org/10.5281/zenodo.5176264). Scripts used to generate and analyze the simulations are available in an open public repository (doi.org/10.5281/zenodo.5032419).

For manuscripts utilizing custom algorithms or software that are central to the research but not yet described in published literature, software must be made available to editors and reviewers. We strongly encourage code deposition in a community repository (e.g. GitHub). See the Nature Portfolio guidelines for submitting code & software for further information.

Data

Policy information about availability of data

All manuscripts must include a data availability statement. This statement should provide the following information, where applicable:

- Accession codes, unique identifiers, or web links for publicly available datasets
- A description of any restrictions on data availability
- For clinical datasets or third party data, please ensure that the statement adheres to our policy

All permeability coefficients generated during this study are included in this published article and its supplementary information files. Minimal source data and materials used in the analysis are available in public repositories https://doi.org/10.5281/zenodo.6010416 for MD simulations, and https://github.com/jacopofrallicciardi/Membrane-permeability-Source-data for experimental measurements.

Field-specific reporting

Please select the one below that is the best fit for your research. If you are not sure, read the appropriate sections before making your selection.

☒ Life sciences ☐ Behavioural & social sciences ☐ Ecological, evolutionary & environmental sciences

For a reference copy of the document with all sections, see nature.com/documents/nr-reporting-summary-flat.pdf

Life sciences study design

All studies must disclose on these points even when the disclosure is negative.

Sample size	Sample size was not predetermined based on statistical methods. At least two independent biological replicates were performed for each condition, accordingly to the standards of the field.
Data exclusions	No data were excluded from the analysis.
Replication	For stopped-flow measurements, multiple acquisitions of each condition were taken for noise reduction. For each lipid mixture, at least a second biological replicate was performed. Each attempt of replication was successful.<br>For dynamic light scattering (DLS) measurements, 10 acquisition were performed. For differential scanning calorimetry (DSC), each sample was scanned 5 times to ensure the reproducibility of the endotherms. For both DLS and DSC measurements, all attempts of replication were successful.
Randomization	Randomization is not relevant for our study as no animal or human subjects were used in the study.
Blinding	The investigators were not blinded during this study as the results are quantitative. Thus, subjective interpretation was not required.

Reporting for specific materials, systems and methods

We require information from authors about some types of materials, experimental systems and methods used in many studies. Here, indicate whether each material, system or method listed is relevant to your study. If you are not sure if a list item applies to your research, read the appropriate section before selecting a response.

Materials & experimental systems

n/a	Involved in the study	
☒	☐	Antibodies
☒	☐	Eukaryotic cell lines
☒	☐	Palaeontology and archaeology
☒	☐	Animals and other organisms
☒	☐	Human research participants
☒	☐	Clinical data
☒	☐	Dual use research of concern

Methods

n/a	Involved in the study	
☒	☐	ChIP-seq
☒	☐	Flow cytometry
☒	☐	MRI-based neuroimaging
